# Back to the Future: Spatiotemporal Determinants of NK Cell Antitumor Function

**DOI:** 10.3389/fimmu.2021.816658

**Published:** 2022-01-10

**Authors:** Joey H. Li, Timothy E. O’Sullivan

**Affiliations:** ^1^ Department of Microbiology, Immunology, and Molecular Genetics, David Geffen School of Medicine at the University of California, Los Angeles (UCLA), Los Angeles, CA, United States; ^2^ Medical Scientist Training Program, David Geffen School of Medicine at the University of California, Los Angeles (UCLA), Los Angeles, CA, United States

**Keywords:** tumor microenvironment, NK cell, immunotherapy, adoptive cell immunotherapy, solid tumor, innate lymphoid cell (ILC)

## Abstract

NK cells play a crucial role in host protection during tumorigenesis. Throughout tumor development, however, NK cells become progressively dysfunctional through a combination of dynamic tissue-specific and systemic factors. While a number of immunosuppressive mechanisms present within the tumor microenvironment have been characterized, few studies have contextualized the spatiotemporal dynamics of these mechanisms during disease progression and across anatomical sites. Understanding how NK cell immunosuppression evolves in these contexts will be necessary to optimize NK cell therapy for solid and metastatic cancers. Here, we outline the spatiotemporal determinants of antitumor NK cell regulation, including heterogeneous tumor architecture, temporal disease states, diverse cellular communities, as well as the complex changes in NK cell states produced by the sum of these higher-order elements. Understanding of the signals encountered by NK cells across time and space may reveal new therapeutic targets to harness the full potential of NK cell therapy for cancer.

## Introduction

Cancer is a collection of dynamic diseases that arise from unique tissue types and exist in different stages over time, frequently across multiple anatomical sites in advanced stages. From the primary tumor to metastases, immune status also varies over disease progression and location. Furthermore, the cellular communities present within the tumor microenvironment (TME) likely evolve over time and space. Contemporary studies have focused on antitumor immunity at a single point in time, but evidence from comparisons of tumor-infiltrating immune populations isolated from different tumor stages suggests that differential developmental and functional immune cell phenotypes change during disease progression, the basis of which is not yet understood (1). While many of the cell-cell interactions driving immune dysfunction in the TME have been elucidated to reveal novel immunotherapeutic targets, the mechanisms are poorly contextualized in the framework of spatially and temporally dynamic cancer states.

Natural killer (NK) cells are a subset of cytotoxic group 1 innate lymphoid cells (ILCs) which have the innate ability to detect and kill virally infected or malignant cells (2, 3). While NK cells were the first lymphocytes to demonstrate natural killing of tumor cells, they have remained relatively understudied compared to the intense focus on cytotoxic T cell therapy for cancer treatment (4, 5). However, the clinical relevance of NK cells in cancer is well-established. Increased NK cell abundance has been shown to correlate with better prognosis in multiple solid tumor types (6). Additional studies have also correlated NK cell tumor infiltration, activation and developmental status, and cytotoxic capacity with enhanced antitumor activity (7–11). NK cells have proven to be critical in the initial activation of dendritic cells (DCs), enhancing tumor neoantigen presentation to effector T cells and boosting immune checkpoint blockade (ICB) efficacy by reinvigorating tumor-infiltrating T cells (12). Therapeutic approaches directly utilizing antitumor NK cells have shown promise as well. A growing number of clinical trials are bringing NK cells to the bedside using approaches ranging from chimeric antigen receptor (CAR)-NK cells to bi- and tri-specific killer engagers (BiKEs and TriKEs), all of which have demonstrated the safety and potential efficacy of NK cells as immunotherapy in both solid and hematologic tumor contexts (13). Unleashing the full potential of these therapies will require developing strategies to assist NK cells in navigating the complex immunosuppressive mechanisms of the TME. While NK cells may encounter different immunosuppressive signals than tumor-infiltrating T cells, recent studies suggest that there is overlap between immune checkpoint pathways in the two cell lineages. However, the specific microenvironmental influences, signaling pathways and downstream effects merit further study in cell- and tissue-specific contexts to fully release the therapeutic potential of NK cells in the immunosuppressive tumor milieu (14–16).

When viewed through the lens of heterogeneous tumor architecture, temporal disease states, and diverse and dynamic cellular communities, we can begin to understand the complex changes in NK cell states produced by the sum of these elements. Both preclinical and clinical evidence point to a paradigm of continuous changes in NK cell development and activation over the course of cancer progression (1, 17, 18). Comparisons of NK cell states within the TME from early to advanced disease may provide insight into specific mechanisms that can be targeted to restore antitumor NK cell function in advanced cancers. Furthermore, as our understanding of ILC heterogeneity expands, we will be able to better interpret studies describing the complex array of NK and ILC states present in the tumor that can dictate antitumor responses. Here, we describe the complex dynamics of cancer progression through different stages, tissues, and treatments as they relate to NK cell function. We also outline the network of tumor-intrinsic and NK cell-intrinsic changes during cancer that can alter effective NK cell antitumor function, as well as new approaches available to better study the sum of these interactions. A holistic understanding of the heterogeneous physical, spatial, and temporal interactions affecting NK cells in the TME may reveal new immunotherapeutic targets and engineering approaches to fully unleash the potential of NK cell therapy in cancer.

### Key Variables in Tumor State-Specific NK Cell Function

**Figure 1:**
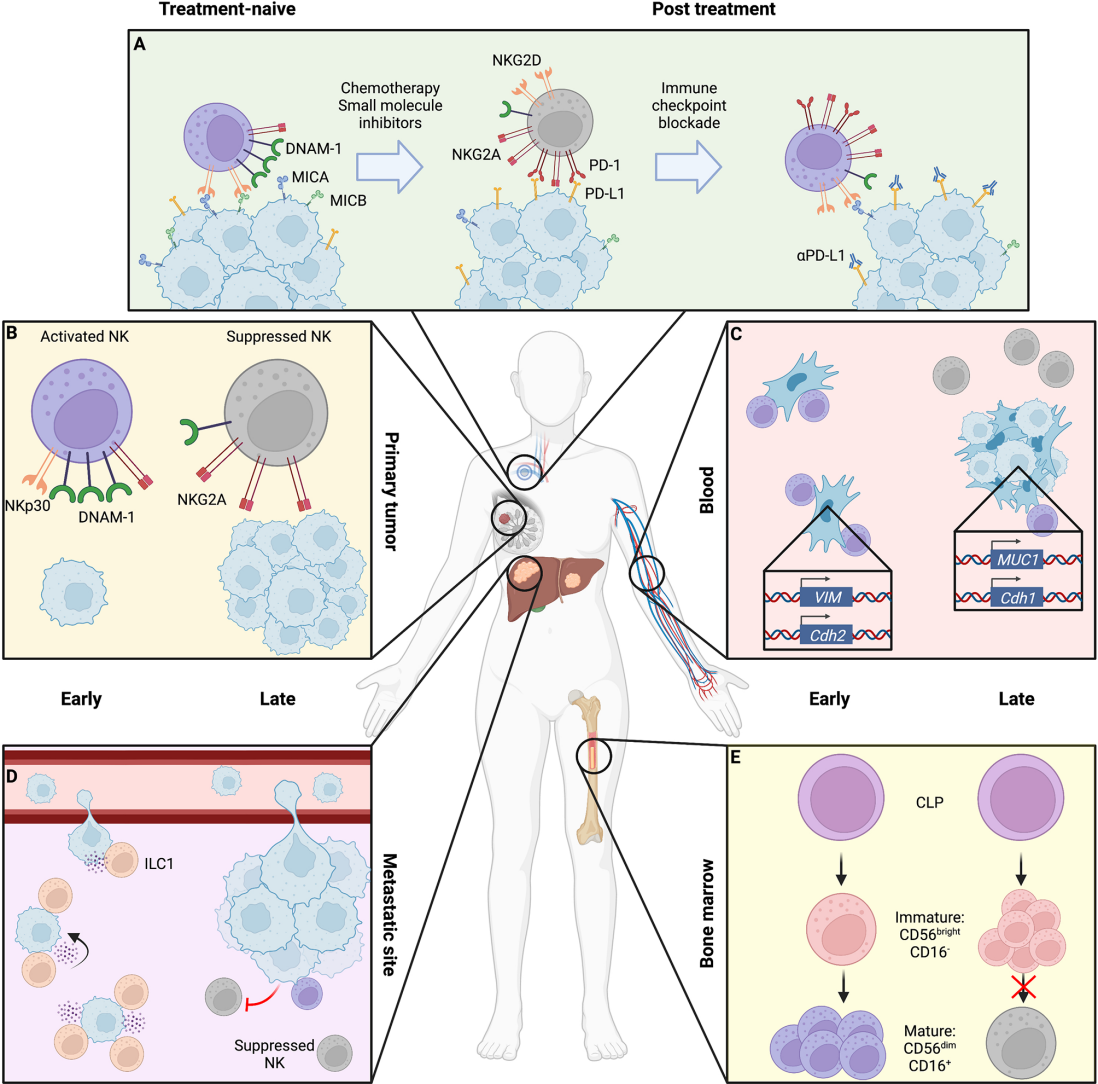
Location- and stage-dependent changes in NK cell status and function. Magnified bubbles represent cancer therapy **(A)**, primary breast tumor **(B)**, peripheral blood with circulating tumor cells (CTCs) **(C)**, liver metastasis **(D)**, and bone marrow niche **(E)**. **(A)** During treatment, cytotoxic chemotherapy and small molecule inhibition can induce NK cell resistance through downregulation of NK activating ligands and upregulation of immune checkpoint molecules on the tumor surface, coupled with a shift in NK cell receptor repertoire toward inhibitory signaling. These may be overcome by sequential immune checkpoint blockade to release suppressed NK cells. **(B)** The late-stage primary tumor induces immunosuppressive changes in NK cells, causing reduced expression of activating receptors like DNAM-1 and NKp30 as well as cytotoxic effector molecules perforin and granzyme, while upregulating inhibitory receptors such as NKG2A. **(C)** In the blood, early-stage CTCs that have fully undergone epithelial-to-mesenchymal transition (EMT) expressing N-cadherin and vimentin are efficiently targeted by NK cells which are overcome by NK-suppressive mixed epithelial-like CTCs retaining mucin or E-cadherin expression in late disease. **(D)** At metastatic sites, ILC1s and NK cells may cooperate to respectively inhibit metastatic seeding and kill tumor cells within established lesions, though by late disease cytotoxic cells are suppressed toward an immature, dysfunctional state. **(E)** NK maturation in the bone marrow niche may be suppressed by tumor presence, arresting NK cells in an immature state. Created using Biorender.com.

While commonly conceptualized as a single homogenous mass, tumors are composed of genetically heterogenous clones, and disease progression takes place over time and space. Moreover, patients receiving NK cell-based therapies will likely have already undergone multiple cycles of cytotoxic and immune modulating therapies, which may alter the activation or developmental status of the patients’ NK cells. We will refer to these broad influences of cancer progression as the “tumor state”; which includes cancer stage, prior therapies, architecture, and anatomy (**Figure 1**).

#### Cancer Stage

Staging is a clinical definition based on the tumor size, location, and degree of spread throughout the body. Solid malignancies are staged by the tumor-node-metastasis (TNM) system, which assigns a numerical value to the tumor size, local and regional lymph node involvement, and distant metastasis (19). TNM classifications are then grouped into stages 0-IV. While the specific tumor size and lymph node involvement criteria vary for different cancer types, in general, stage 0 describes a non-invasive tumor such as ductal carcinoma in situ; stage I and II disease are confined to the primary site or involve local lymph nodes; and stage III and IV describe disease that has spread to distant lymph nodes or metastasized to other organs, respectively. Intrinsic to this model are progressive changes to the TME as tumors gain the capacity to grow larger, evade immunosurveillance and therapy, and disseminate. Relatively few studies examine the temporal changes to the functional and developmental status of antitumor immune cells, while ICB is largely still reserved for later stage advanced, unresectable, or relapsed disease in the clinic.

NK cells isolated from invasive breast cancer patients exhibited an altered phenotype and function compared to NK cells from patients with noninvasive carcinoma *in situ* (NCIS). Comparison between healthy donor, benign mammary tumor, NCIS, and localized, locally advanced, and metastatic invasive breast cancer peripheral blood NK cells displayed progressively decreased expression of activating receptors NKp30, NKG2D, 2B4, DNAM-1, CD16, and NKp46, as well as increasing expression of inhibitory NKG2A and CD85j/LIR-1 (20). Functionally, peripheral NK cells from late-stage disease displayed decreased cytotoxicity and interferon-γ (IFN-γ) production compared to peripheral NKs from patients with benign or noninvasive tumors. Tumor tissue was enriched in immature CD56^bright^ NK cells compared to peripheral blood or paired normal mammary tissue from the same patient. Compared to peripheral blood, tumor-infiltrating NK cells expressed lower levels of activating receptors and had decreased cytotoxic function *in vitro*. Notably, the premalignant NK cell phenotype was restored in patients who had undergone surgical resection of breast cancer and not relapsed for at least 5 years after surgery. These data suggest that as the tumor state progresses, there is progressive yet reversible inhibition of NK cell development and terminal maturation in both the tumor and periphery. These findings are consistent with evidence from mouse models that tumor burden leads to impairments in peripheral NK cell maturation (21). However, this study did not compare intra-tumoral NK cells between NCIS and invasive carcinoma, as the authors noted low lymphocyte yields from patient tumor samples. Future studies using single cell approaches may better resolve differences in NK cell profiles between tumor stages and detect distinct subtypes. Similar stage-specific impairment of NK cells has been seen in melanoma, as peripheral NK cells from treatment-naïve stage IV melanoma patients displayed impaired degranulation in tumor co-culture compared to NK cells isolated from earlier stage patients (22). Additionally, there was overall increased heterogeneity in NK cell expression of activating receptors NKp46, NKp30, NKG2D, and NKp44 in patients compared to healthy donors. Stage III and IV patients with locally advanced or metastatic disease had higher variance in HLA-DR expression. NKp46 expression overall decreased as disease stage progressed, while higher expression in specific patients correlated with prolonged survival. Together, these suggest that peripheral NK cell impairment in melanoma progresses in a stage specific manner. However, while peripheral NK cell activation status may have important implications for control of systemic metastasis, particularly in late-stage patients, comparison of intra-tumoral NK cells from resected tumors may provide deeper insight into TME-specific immunosuppression.

#### Treatment History

Although immunotherapy has come to the frontline in an increasing number of tumor types depending on tumor expression of immune checkpoint molecules, ICB is frequently used as an alternative treatment for patients with advanced disease (23, 24). Prior chemotherapy, small molecule inhibitor treatment, or immunotherapy can significantly impact the local and systemic immune landscape. Coupled with highly individualized patient treatment histories, understanding the effects of prior therapy is a crucial aspect of translating studies of tumor immune contexture to the realities of patient care.

Small molecule inhibitors are the mainstay of treatment for multiple cancers (25). Targeted inhibitors first entered clinical use for hematologic malignancies in the form of tyrosine kinase inhibitors like imatinib and dasatinib (26). NK cell lymphocytosis has been reported as a marker of favorable response to dasatinib treatment in patient with chronic myelogenous leukemia (CML) (27). However, studies investigating the functional effects of dasatinib on NK cells have revealed discordant results. A study of 8 CML patients undergoing dasatinib treatment revealed significantly elevated NK cell levels after treatment, while *in vitro*, NK cells isolated from patients who experienced lymphocytosis exhibited increased cytotoxicity in a ^51^Cr release assay (28). These results were supported by studies indicating increased NK cell proliferation and cytotoxicity after *in vitro* dasatinib treatment of healthy human peripheral blood cells (27). Another study of peripheral blood from CML patients suggested that dasatinib treatment, but not imatinib or nilotinib, induces long-term suppression of inhibitory NKG2A on NK cells with accompanying increased cytotoxicity (29). Conversely, *in vivo* dasatinib treatment in mice resulted in decreased NK cell clearance of MHC class I-deficient RMA-S tumor cells (30). Direct comparison of these results may be confounded by differences in patient histories, dosing, and preclinical models, but further study may reveal the underlying variables that dictate the observed variability in NK cell activation or inhibition after dasatinib exposure. In melanoma, inhibitors of the common driver mutations MEK and BRAF are now frequently used in combination with ICB (31, 32). Similar to the varying effects of tyrosine kinase inhibition, there is conflicting evidence surrounding the effects of BRAF inhibition on NK cell sensitivity. One preclinical study suggested that vemurafenib-resistant melanoma cell lines were more sensitive to NK cell killing, while another study found that vemurafenib treatment led to NK cell resistance *via* decreased tumor expression of activating ligands (33, 34). While inconsistent, these data suggest that BRAF inhibition significantly modifies tumor cell interactions with NK cells and merit deeper consideration in the context of patient treatment. Other small molecules such as MEK inhibitors have been suggested to exhibit broadly proimmunogenic roles, suggesting potential for combination therapy with immunotherapy (35).

Traditional cytotoxic chemotherapy can have varied effects on the immune system due to its ability to target all rapidly-dividing cells (36). At standard doses, chemotherapy may activate antitumor immunity through induction of immunogenic cell death or modulation of tumor-intrinsic immune escape mechanisms. Low immune-modulating doses of chemotherapy can also be used to deplete specific subsets of immunosuppressive cells, for example low-dose cyclophosphamide for regulatory T cell (Treg) depletion. NK cells from stage IV melanoma patients who have undergone cytotoxic chemotherapy with dacarbazine, fotemustine, cisplatin, vincristine, or cyclophosphamide displayed heightened expression of the inhibitory receptor NKG2A as well as the activating receptor NKp46 (37, 38). Prior to treatment, NK cells were found to be NKp46^lo^NKG2A^lo^ but converted to an NKp46^hi^NKG2A^hi^CD158a^lo^ phenotype after chemotherapy administration. However, NK cells derived from patients treated with chemotherapy had decreased cytotoxic functionality in coculture, potentially due to increased inhibitory NKG2A signaling. Preclinical studies indicated that cisplatin-resistant non-small cell lung cancer (NSCLC) cell lines were resistant to NK cell killing *via* upregulated programmed death ligand 1 (PD-L1) and decreased NKG2D ligand expression (39). Treatment with anti-PD-L1 ICB and MEK inhibition synergized to restore NK cell sensitivity. Consistent with these results, biopsies from NSCLC patients who had received neoadjuvant cisplatin revealed increased PD-L1 expression after cisplatin treatment, while anti-PD-L1 treatment in mouse models *in vivo* effectively inhibited tumor growth (40). Together, these data indicate that prior chemotherapy can drastically modify tumor susceptibility to NK cell cytotoxicity.

ICB, though initially developed to prevent cytotoxic T cell inhibition, has been shown to affect NK cells. Mouse NK cells activated *in vitro* upregulated the stimulatory receptor CD28 and immune checkpoint CTLA-4, which both compete for binding to ligands CD80 and CD86, and responded to receptor engagement by increasing or decreasing IFN-γ production, respectively (41). NK cells expressing CD28 and CTLA-4 were also identified in mouse RMA-S, melanoma, and lung tumors, and responded to CTLA-4 activation *ex vivo* with decreased IFN-γ production (14). Furthermore, ipilimumab (a CTLA-4 blocking antibody) efficacy in melanoma patients positively correlated with increased CD56 transcript expression in post-treatment tumor biopsies (42). In another cohort of ipilimumab-treated advanced melanoma patients, NK cells shifted toward a mature CD56^dim^ phenotype expressing increased NKp46 and TIM-3 and decreased inhibitory killer Ig-like receptors (KIRs). Both CD56^bright^ and CD56^dim^ NK cells expressed increased CD16 and PD-1. Thus, as immunotherapy increasingly becomes a frontline treatment option and patients begin to experience sequential rounds of ICB, the effects of prior immunotherapy on NK cell status will need to be considered for future immunotherapy or cell therapy efficacy. Additionally, clinical trials combining cytotoxic chemotherapy and ICB further support the nuances of cumulative treatment effects on antitumor immunity. Combined ipilimumab and dacarbazine therapy for stage IV melanoma patients yielded increased survival compared to dacarbazine alone (43). Moreover, in both NSCLC and small-cell lung cancer (SCLC), sequential cycles of paclitaxel and carboplatin chemotherapy followed by chemotherapy plus ipilimumab were superior to the reversed sequence of ICB followed by chemotherapy, suggesting that prior chemotherapy can play a clinically significant role in immune priming (44, 45). Deeper elucidation of how NK cells respond and interface with adaptive immunity after combined chemotherapy, small molecule inhibitor, and ICB treatment will help guide logically designed combination therapy regimens.

#### Anatomical Location

While NK cells can be found in solid tumors, the absolute level of NK cell infiltration into the TME varies drastically by primary tumor site. In a comparison of tumor-infiltrating NK cell quantity across tumor types, tumors with the highest infiltration of NK cells included acute myeloid leukemia (AML), diffuse large B-cell lymphoma (DLBCL), and testicular germ cell tumors, while CRC, breast cancer, and melanoma exhibited low NK infiltration (46). These data suggest that there are tissue-specific determinants of NK cell infiltration in tumors, and studies of healthy tissue may guide our understanding of tumor-specific NK cell modulation. For example, mucosal epithelium exposed to the external environment, such as the lung and gut, or organs that encounter high blood flow like the liver have been found to contain specialized ILC subtypes as well as the ability to preferentially attract circulating NK cells (47, 48). This may be driven by specific inflammatory signals encountered at these different sites. The lung alveoli are constantly exposed to pathogens and external toxins and therefore demand a significant innate immune defense, provided partly in the form of lung-resident NK cells as well as increased migration of circulating NK cells into the tissue during infection (47). Recruited circulating NK cells have been found to express upregulated CD49a as well as increased IFN-γ and perforin (49). Similarly, liver-resident NK cells may be exposed to the constant flow of pathogens in blood filtering through hepatic sinusoids, whereby ligands uniquely expressed in the hepatic sinusoidal microenvironment maintain the population of liver-resident NK cells through activation of CCR5 and CXCR6 (47). Understanding similar factors that are unique to tumors based on organ of origin may reveal targets to modulate NK cell attraction and function within the tissue.

Additionally, multiple studies have indicated the existence of organ-specific ILCs and tissue-resident NK cell (trNK) populations in normal physiology (47, 50). For example, liver-specific type 1 ILCs (ILC1s) have been identified based on expression of CD200r1 and CD49a with dependence on the transcription factor Hobit for terminal maturation into cytotoxic effector cells (51, 52). Thus, it is possible that the signals responsible for tumor-type-specific ILC variation overlap with the mechanisms governing ILC specificity in healthy tissue. Indeed, unique populations of cytotoxic tumor-infiltrating ILCs have been identified in the polyoma middle T virus (PyMT) mouse model of spontaneous breast cancer (18). Tissue-specific differences in intra-tumoral NK infiltration therefore most likely differ not only in absolute quantity of NK cells but also in specific ILC subtype, and further study will be needed to identify the determinants of both.

NK cells play a critical role in metastatic control (53). In patients, metastatic load has been found to inversely correlate with quantity of both circulating peripheral and intra-tumoral NK cells in multiple cancer types (53). In hepatocellular carcinoma (HCC), the abundance of IFN-γ -responsive peripheral NK cells was predictive of recurrence risk after curative surgery or radiotherapy, suggesting that circulating NK cells actively contribute to control of residual circulating tumor cells (CTCs) with metastatic capacity after resection or resolution of the primary tumor (54). It is likely that NK cells and other ILCs exert anti-metastatic effects at different stages of the metastatic cascade, for example both controlling CTC dissemination in the blood and surveilling the site of metastatic seeding in tissues. Indeed, a recent study found that liver metastases in a mouse model were monitored by both circulating NK cells as well as tissue resident ILC1 (55). In this study, the authors found that ILC1 primarily served to limit metastatic seeding, while NK cells infiltrated metastatic nodules to exert cytotoxic effects within established lesions. The metastatic niche was reciprocally able to educate infiltrating NK cells toward unique populations of immature CD49a^-^Eomes^-^ and cytotoxic CD49a^+^Eomes^+^ phenotypes in a tumor type-dependent manner. With greater understanding of metastatic control by NK cells in spatial and temporal contexts, adoptive NK cell therapy could potentially be used as an adjuvant therapy to control micrometastases or CTCs after resection of the primary tumor. Conversely, neoadjuvant NK cell therapy for unresectable advanced disease may have the capacity to specifically reduce metastatic burden so that surgical treatment becomes a viable option.

During tumor progression, CTCs are thought to seed metastatic sites on a microscopic level. CTCs have been proposed to fall into monoclonal or polyclonal subsets, whereby monoclonal CTCs have undergone epithelial to mesenchymal transition (EMT) and exist primarily as isolated tumor cells in the blood, while polyclonal CTCs retain some epithelial characteristics, allowing them to traverse the vasculature in clusters (56). A recent study suggested that single CTCs that have fully undergone EMT were more susceptible to NK cell killing due to upregulation of NKG2D ligands and downregulation of NK-inhibiting HLA molecules, while polyclonal epithelial-like CTCs were resistant and more efficiently seeded metastases (57). Supporting this hypothesis, it has been shown that NK cells can more efficiently kill mesenchymal-like cancer cells compared to more differentiated epithelial-like cells that had not undergone EMT (58). NK cells may also suppress metastasis by maintaining disseminated tumor cells in a dormant state, as local deactivation of NK cells by activated hepatic stellate cells (aHSCs) resulted in release of dormant p27^+^ tumor cells in the liver and subsequently increased metastatic outgrowth (59). Local NK suppression was determined to be mediated by CXCL12 released by aHSCs interacting with CXCR4 on the NK cell surface. Maintenance of tumor dormancy was found to be dependent on NK cell production of IFN-γ. Together, these suggest that NK cells are important for multiple steps of the metastatic cascade, and targeted application of NK cells to control disease spread may prove useful for treatment of cancers like PDAC that frequently present late in disease course.

#### Tumor Microenvironment Structure

Tumors are constructed of diverse structural parameters that affect immune function. Spatial immune infiltration has become a factor of interest for immunotherapy of solid tumors. Solid tumors have been described to exhibit both inter- and intra-tumoral spatial heterogeneity in multiple studies (60–63). One classification schema sorts areas into immune inflamed, immune excluded and immune desert, with unique molecular features to each spatial subtype (64). However, categorization of tumors based on immune infiltration to this point has focused heavily on differential T cell infiltration, with less emphasis on the spatial heterogeneity of other cell types (65). The precise spatial distribution of NK cells in patient tumors is poorly examined in the literature, though studies have confirmed that NK cells are able to localize to tumor tissue (61, 66). A study using multiplex IF staining of tissue microarrays from periampullary adenocarcinomas revealed that CD56^+^NKp46^+^ NK cells tend to be confined to the stromal compartment rather than infiltrating tumor nests (61). Similarly, studies of renal cell carcinoma (RCC) tumor microarrays also identified NK cells as excluded to the peri-tumoral region along with both helper and cytotoxic T cells (67). High grade tumors were found to contain more peri-tumoral granzyme B^+^ NK cells, while tumors containing more LAG3^+^TIM3^+^ exhausted T cells tended to also contain more intra-tumoral and peri-tumoral granzyme B^+^ NK cells. These data point to the complex spatial interactions that NK cells engage in the TME, and applying similar spatial approaches to other tumor types may reveal clinically relevant local immunosuppressive cell communication networks.

In many tumors, stroma makes up a major portion of tumor tissue. While stromal cells play a key role in immunosuppression, the buildup of fibrotic tissue, termed desmoplasia, is also thought to have a significant impact on tumor progression (68–70). In breast cancer, the tumor stroma to immune infiltration ratio was found to be a significant predictor of recurrence-free period (71). 87% of patients with high levels of infiltrating cytotoxic T cells and NK cells combined with low density of tumor stroma were recurrence free 10 years post tumor resection, whereas only 17% of patients with low immune infiltrate and high stroma remained free of disease recurrence. Likewise, a TME risk score calculated for gastric cancer based on high/low NK cell infiltration and high/low stroma was predictive of chemotherapy response and survival in gastric cancer patients (7). Patients with tumors that had high NK cells and low stroma had the best prognosis while NK low/stroma high patients had the worst outcomes of the studied group. While this study identified NK cell and tumor stromal abundance as independently prognostic factors, they could also be linked through direct effect of NK cells on fibrosis. In the liver, NK cells have been shown to have a direct antifibrotic role, though their activity is antagonized by aHSCs. NK cells have been suggested to attenuated diet- or carbon tetrachloride-induced liver fibrosis in mice in an IFN-γ-dependent manner, and antibody-mediated blockade of NKG2D or TRAIL abrogated NK cell antifibrotic activity (72). In hepatitis B and C patients with liver cirrhosis, aHSCs were able to undergo emperipolesis with antifibrotic NK cells in a TGF-β manner, creating cell-in-cell structures in which NK cells underwent apoptosis (73). It has yet to be determined if NK cells display antifibrotic activity within desmoplastic tumors. Furthermore, considering the previously described dynamics of aHSC-metastatic niche formation and antimetastatic NK cell function, it will be interesting to examine the interplay between fibrosis, metastatic seeding, and NK cell surveillance.

As tumors grow, the accompanying growth of new vasculature is essential for nutrient delivery to rapidly-proliferating tumor cells (74). Tumor vasculature and lymphatics play multiple clinically relevant roles: vessels supplying the tumor serve as entry points for intravenously administered chemotherapies and biologics, avenues for trafficking immune cells and conduits for CTCs leaving the primary tumor to seed metastases. Tumor vasculature is known to develop in a disordered manner due to its arising from an overload of pro-angiogenic factors such as vascular endothelial growth factor (VEGF) rather than being guided through the normal process of vasculogenesis. As such, tumor vasculature is leaky, tortuous, and fragile (75). Over disease development, poorly perfused solid tumor cores often resultantly become acidic, hypoxic, and necrotic, with significant effects on immune function within the TME. Local acidity and hypoxia-induced molecules such as hypoxia-inducible factor 1-α (HIF-1α) have significant suppressive effects on NK cells, as previously reviewed (2). Antiangiogenic therapy targeting pro-angiogenic molecules have become a useful therapy for certain diseases, such as bevacizumab for glioblastoma multiforme (GBM) (76). Clinical trials combining bevacizumab with anti-PD-L1 therapy have shown promise in multiple studies (77). Considering the evidence that ICB both modulates and relies on NK cells for full effect, it is likely that further study will implicate NK cells in the efficacy of these anti-angiogenic approaches.

Tertiary lymphoid structures (TLSs) are histologically identifiable structures within peripheral tissues that are thought to serve as sites of lymphoid development and activation functionally similar to secondary lymphoid organs (SLOs) like the spleen. Tumor TLSs have drawn interest as a potential biomarker of response to immunotherapy or even as a concentrated source of potentially inducible antitumor immunity (78). Increased density of TLSs in biopsies was prognostic of better outcomes in multiple solid tumor types independent of stage (78). Interestingly, TLSs appear to be primarily populated by B and T cells and are devoid of NK cells, yet TLS presence correlates with higher non-TLS CD57^+^ NK cell infiltration in oral squamous cell carcinoma (79). The mechanism of association between NK cell-devoid TLSs and NK cell infiltration has yet to be characterized but may provide further insight into how NK cells proceed to activate adaptive antitumor immunity. Notably, maintenance of TLSs in influenza infection required DC-derived lymphotoxin β (LTβ), CXCL12 and 13, and CCL19 and 21, while the presence of mature DCs in tumor TLSs was associated with increased effector-memory T cells and patient survival. These data suggest that DCs may be important for the maintenance and function of tumor TLSs, potentially *via* initial recruitment by the recently described NK-DC axis (12, 80, 81).

Unique molecular features in different tumor types may also mold the development of specific immune environments. Solid tumors have been extensively molecularly subtyped, and multiple cancers have been identified as harboring a unique common oncogenic driver (82–84). For example, >90% of pancreatic ductal adenocarcinomas (PDAC) are driven by mutant KRAS, while ~50% of melanomas house BRAF driver mutations (24, 85). Specific oncogenes can induce differential inflammatory states that may alter NK cell and other immune dynamics within the tumor (86). Oncogenic KRAS may be pro-inflammatory *via* induction of IL-6, CCL5, NF-kB, and other immune modulating molecules (87). Similarly, mutant BRAF in melanoma stimulates MAPK signaling and can result in expression of IL-6 and IL-10, promoting immune-tolerant DC maturation and inhibiting cytotoxic T cells (88). Thus, tumor-intrinsic oncogenes and mutations could represent critical upstream mediators regulating NK cell suppressive ligands expressed on multiple cell types in the TME through tumor-derived factors (87). Immunosurveillance can additionally be influenced by varying degrees of mutational load, microsatellite instability, and copy number alterations between tumors (89). To add to the complexity, the prevalence of certain molecular features within a patient’s disease evolves during disease progression, likely as a product of clonal selection through treatment and immunoediting (90). Specific enrichment for intrinsically less immunogenic and more immunosuppressive tumor clones in late-stage disease may partly explain the progressive NK cell dysfunction described in previous sections. If this is true, immunotherapy at earlier timepoints as well as rational combination treatment with immunotherapy and small molecule inhibitors of driver oncogenes may prove to be useful therapeutic strategies.

### Cell-Cell Interactions in the Tumor Microenvironment

**Figure 2:**
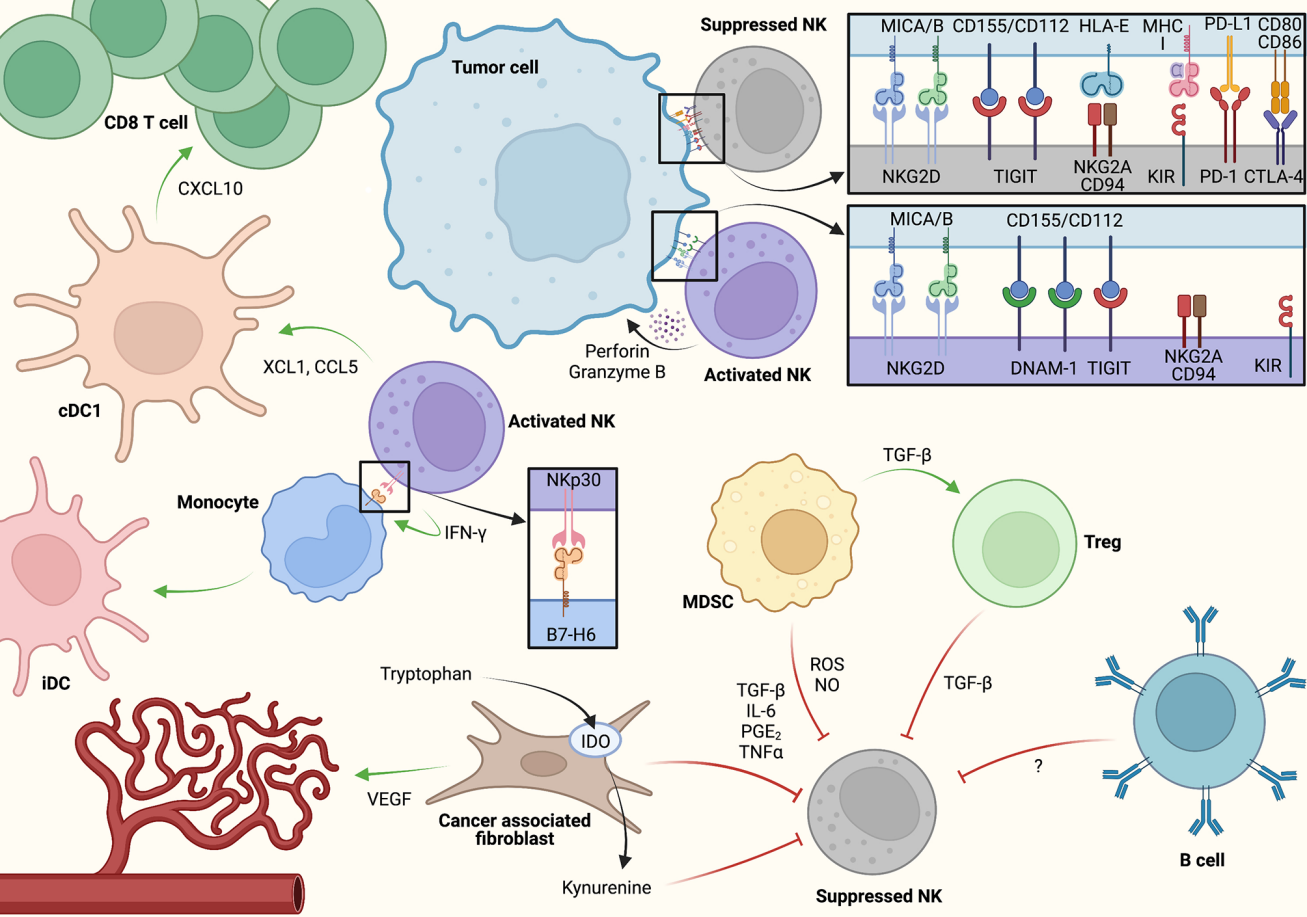
A model for cell-cell interactions and key signaling molecules associated with NK cell activation and inhibition within the tumor microenvironment (TME). NK cells may induce adaptive antitumor immunity *via* direct conventional dendritic cell (cDC1) activation as well as through monocyte polarization toward inflammatory dendritic cell (iDC). At the same time, immunosuppressive crosstalk between cancer-associated fibroblasts (CAFs), myeloid-derived suppressor cells (MDSCs), regulatory T cells (Tregs), and potentially B cells/B regulatory cells through soluble factors restrain antitumor NK cell activity. Created using Biorender.com.

The importance of the interactions between tumor and non-tumor cells that make up the TME has become increasingly clear in both research and clinical settings. Rather than focusing solely on tumor cell-intrinsic signaling, recent efforts have focused on diverse cellular components in the TME and how they interact to promote or arrest immune tumor surveillance and tumor growth (91–95). As NK cell activation is determined by the combination of activating and inhibiting signals from their environment, the cell-cell interactions that NK cells encounter in the TME have a direct impact on their functionality (**Figure 2**). While the abundance of specific ligand-receptor interactions likely varies based on key variables influencing NK cell activation within the tumor as discussed previously, we will discuss notable cell type-specific interactions which influence NK cell function in the TME.

#### Tumor Cells

Transformed malignant cells are natural targets for NK cells due to tumor expression of stress ligands such as MICA/MICB that interact with NKG2D on the NK cell surface (96). MHC class I normally inhibits NK cells by binding to inhibitory killer Ig-like receptors (KIRs) (96). NK cells can be activated by cells which have lost MHC class I, such as tumor cells utilizing MHC downregulation as an escape mechanism (96). Tumor cells have also evolved other methods to escape NK cell killing by expression of other inhibitory ligands. For example, tumor cells can express CD155 which interacts with both the activating receptor DNAM-1 and inhibitory receptor TIGIT on NK cells. Within the tumor, CD155 is largely overexpressed by tumor cells, though it can be also expressed on myeloid-derived suppressor cells (MDSCs) (97). Paradoxically, CD155^+^ primary ovarian carcinoma cells have been found to decrease DNAM-1 expression on NK cells through direct contact, rendering NK cells less responsive to DNAM-1 activation (98). Another study demonstrated that membrane CD155 triggers DNAM-1 internalization and degradation, decreasing DNAM-1 on the NK cell surface and pushing the receptor balance toward inhibitory interactions with TIGIT (99). Tumor cells may also produce soluble CD155 to inhibit DNAM-1^+^ NK cells without direct contact (100). In metastatic melanoma, high tumor expression of CD155 was predictive of poor response to anti-PD-1 treatment (101). Furthermore, CD112 on the tumor surface has been found to function similarly to CD155, downregulating DNAM-1 and inhibiting NK cell activation, highlighting CD112 as another potential clinically relevant target (102). HLA-E, a noncanonical human leukocyte antigen (HLA) molecule, has also been shown to inhibit NK cell killing *in vitro via* interaction with the NKG2A/CD94 heterodimer found on the NK cell surface (103). HLA-E was identified as a significant negative regulator of tumor NK cell sensitivity in combined analysis of two high-throughput screening methods (104). Inhibition of NKG2A expression in another study allowed NK cells to regain cytotoxic function against HLA-E^+^ tumor cells (105). In patient tissues, HLA-E was found to be highly expressed primarily on tumor cells in neoplastic compared to healthy tissue (106). Furthermore, in gynecologic cancers, high HLA-E expression in patients abrogated the survival benefit of high levels of cytotoxic tumor-infiltrating lymphocytes, suggesting that tumor HLA-E expression is a relevant factor when considering ICB or adoptive cell therapies for patients (107). In addition to regulation of NK cell function *via* cell surface interactions, tumor modulation of NK cell function may also be partly due to chromatin alterations. In T cells, epigenetic profiling has been suggested to be a more robust method of subset stratification than transcriptional signatures (108). Accordingly, it is possible that the same is true for NK cells, and that tumor-induced epigenetic states may denote bona fide NK cell subsets. During NK cell development, expression of NKG2A is regulated by DNA methylation status (109). Furthermore, expression of the activating NKG2D receptor as well as subsequent inflammatory function is regulated by histone methylation status *via* Ezh2 histone methyltransferase and Jumonji demethylases such as UTX and JMJD3 (110–112). NK cells differentiated from hematopoietic stem cells treated with an Ezh2 inhibitor exhibited increased antitumor activity, an effect also seen in cytotoxic T cells (113). Furthermore, direct NK cell exposure to breast tumor organoids was found to induce a “resting” NK cell state characterized by increased LAG3, KLRG1, and TIGIT expression as well as downregulation of genes involved in immune cell proliferation, adhesion, and activation (114). Functionally, resting NK cells exhibited decreased cytotoxicity and metastatic control *in vitro* and *in vivo* which was reversed with anti-TIGIT, anti-KLRG1, and DNA methyltransferase inhibitor treatment. Together these studies suggest that tumor cell contact can induce an epigenetically determined and potentially reversible dysfunctional NK cell state which could be leveraged to increase anti-tumor NK cell activity.

#### Stromal Cells

Cancer-associated fibroblasts (CAFs) are an abundant and active population of mesenchymal stromal cells in solid tumors, distinguished from normal tissue fibroblasts primarily by expression of specific markers including α-smooth muscle actin (SMA), fibroblast activating protein (FAP), or fibroblast-specific protein-1/S1001A4 (94). CAFs are prolific producers of soluble factors implicated in tumor immunosuppression such as transforming growth factor beta (TGF-β), indoleamine-2,3-dioxygenase (IDO), prostaglandin E2 (PGE_2_), interleukin-6 (IL-6), tumor necrosis factor a (TNF-α), and VEGF (2). The effects of these molecules on tumor-infiltrating NK cells have been previously reviewed (2). In coculture experiments combining primary healthy donor NK cells with CAFs isolated from metastatic melanoma or healthy skin samples, the presence of CAFs attenuated IL-2-induced NK cell activation and upregulation of activating receptors NKp44, NKp30, and DNAM-1, while also mildly decreasing expression of perforin and granzymes (93). Direct CAF-NK cell contact was required for DNAM-1 inhibition, while NKp44 and NKp30 downregulation was found to be dependent on CAF-derived PGE_2_. While CAFs cultured alone spontaneously released PGE_2_, CAF-NK cell coculture significantly increased CAF PGE_2_ release, suggesting that this may be an immunosuppressive mechanism prevalent within the TME. In the liver, CAFs may develop from aHSCs, a significant component of both normal liver parenchyma and the HCC TME. While the distinction between activated stellate cells and CAFs is not well defined, it has been shown that aHSCs accompanying the formation of liver metastases can secrete the chemokine CXCL12 and induce NK cell quiescence through CXCR4 binding, as previously noted (59). CAFs can also cooperate with other immunosuppressive cells of the TME to alter NK functionality. In colorectal cancer (CRC), CAFs were found to promote tumor-monocyte interactions, polarizing macrophages into an immunosuppressive M2-like phenotype (115). CAFs in CRC were also found to directly inhibit NK cell expression of activating receptors and effector molecules such as CD69, NKG2D, DNAM-1, NKp30, NKp44, perforin, and granzyme B in coculture, suggesting that CAFs in the TME can act both directly and indirectly to mediate NK cell suppression (116).

#### Dendritic Cells

Recently, an NK-DC axis that plays a critical role in tumor surveillance and ICB response has been described (12). In multiple COX-deficient mouse tumor models, conventional type 1 dendritic cell (cDC1) accumulation and subsequent CD8^+^ T cell infiltration was determined to be dependent on the presence of NK1.1^+^CD3^-^ NK cells (12). Mechanistically, XCL1 and CCL5 derived from intra-tumoral NKs recruited cDC1s into the tumor, which then produced CXCL10 to attract T cells. PGE_2_, the production of which depends on COX enzymatic activity, impaired both CCL5 and XCL1 production from activated NK cells and expression of the corresponding chemokine receptors, CCR5 and XCR1, in cDC1s, suggesting an immunosuppressive mechanism that may be therapeutically targeted to boost activation of adaptive immunity. These data additionally augment previous evidence that human NK-DC interactions enhance DC cross-presentation of tumor antigens and provide further mechanistic insight within the TME (117). Furthermore, specific ILC subtypes may preferentially interact with DCs, as a recent study identified a group of ILC1-like NK cells characterized as CD27^+^CD62L^-^CD160^+^ virus-responsive Ly49H^+^ NK cells during mouse CMV (MCMV) infection (118). These cells exhibited transcriptional and functional signatures of both ILC1s and NK cells but interestingly were also marked by high expression of Batf3 and XCL1. ILC1-like NK cells were found to be resident in the spleen and were necessary for preferential clustering of cDC1s to prime antigen-specific CD8^+^ T cells, though it has yet to be determined if these cells were truly tissue-resident or circulating NK cells that had transiently extravasated into the tissue during infection. It will be interesting to study the presence and potential role of these ILC1-like NK cells in the tumor, as these new subtypes suggest that subset-specific NK-DC priming in the TME may provide ways to improve DC-based immunotherapies.

Studies of NK-DC interactions in other inflammatory states further suggest that the function of these cell types are closely linked (119). Human NK cell interaction with CD14^+^CD16^-^ monocytes was found to influence monocyte differentiation into DCs *via* cell-cell interactions stabilized by NKp30 (120). Early on in this process, some monocytes were killed by NK cell cytotoxicity, but remaining monocytes were polarized toward DC differentiation *via* IFN-γ. Early monocyte culling by NK cells seen in this study is also reminiscent of evidence suggesting that cytotoxic NK cells edit the pool of immature DCs to remove less immunogenic precursors and make room for expansion of more immunogenic DCs (121, 122). In these studies, human DCs matured from an NK cell-edited pool exhibited a higher capacity to activate cytotoxic T cells. While these data further support the importance of an NK-DC axis in bridging innate and adaptive immunity, it is likely that unique cytokine milieus within the TME may alter the potency of NK-derived cytokines driving DC activation, and further study could guide the design of adoptive cell-delivered cytokine payloads for local modulation of cytokine balance and DC function. With the understanding of NK-DC-T cell cross-activation in tumor surveillance, one outstanding question is how the timing of these interactions affects optimal antitumor activity. In inflammatory states, NK cells are thought to be rapid responders existing in a poised epigenetic state to facilitate rapid expression of effector chemokines such as IFN-γ (123). If NK cell activation is required to induce the maximal antitumor response from the adaptive immune system downstream, it is possible that NK cells play a primary role in surveillance during early tumorigenesis, after which the responsibility of tumor control transitions to activated cytotoxic T cells in later disease stages. Understanding these dynamics may provide the basis for rational design of immunotherapy regimens that selectively modulate innate or adaptive immunity based on cancer stage.

#### Myeloid Cells

Myeloid cells in the TME exist largely as MDSCs and tumor-associated macrophages (TAMs) (124). MDSCs are subdivided into phenotypically and functionally different polymorphonuclear and monocytic MDSCs (PMN-MDSCs and M-MDSCs, respectively). PMN-MDSCs more commonly populate peripheral lymphoid organs, while M-MDSCs are more commonly found in the tumor itself and promote monocyte differentiation into TAMs. These cells suppress antitumor immunity through a variety of mechanisms, generally involving the production of reactive oxygen species (ROS) or soluble factors similarly to CAFs. The effects of these soluble inhibitory factors on NK cells have been previously reviewed (2). MDSCs can also inhibit NK cells and other immune effector cells through direct contact *via* immune checkpoint molecules or CD155-TIGIT interactions (125, 126). Clinically, MDSC accumulation is associated with disease progression in multiple tumor types, as well as being a prognostic marker in NSCLC, breast cancer, gastrointestinal malignancies, and melanoma (127).

In PyMT mice, TAMs were found to inhibit NK cell cytotoxicity in a TGF-β-dependent manner (128). Coculture with TAMs induced a CD27^-^CD11b^+^ terminally differentiated phenotype. Adoptive transfer of splenic MDSCs from tumor-bearing mice were similarly able to suppress NK cell antitumor function in a proposed contact- and STAT5-dependent manner, suggesting that both intra-tumoral and peripheral MDSCs possess spatially and mechanistically distinct modes of NK cell suppression (129). In another study, TAMs isolated from human gastric cancer had a similar capacity to impair NK cell IFN-γ and TNF-α responses as well as proliferation (130). “Adaptive” human NK cells that exhibit features of memory response to cytomegalovirus (CMV) infection were found to be uniquely resistant to patient-derived MDSC suppression due to decreased TIGIT expression (126). MDSCs increased CD155 and CD112 expression in an ROS-dependent manner, and CD155-TIGIT engagement inhibited ERK and ZAP70 signaling in NK cells. These data appear to support studies in advanced melanoma suggesting that cytokine-induced memory-like NK cells can exhibit superior antitumor function (131). Additionally, TIGIT-blocking ICB may be a promising new avenue toward releasing MDSC inhibition of NK cells in the TME. Indeed, primary analysis of the CITYSCAPE trial examining combined anti-TIGIT antibody tiragolumab plus anti-PD-L1 atezolizumab for locally advanced or metastatic NSCLC suggested a significant improvement in overall response rate and progression-free survival (132). As a significant component of MDSC immunosuppression is derived from crosstalk with other suppressive members of the TME, for example induction of Tregs *via* TGF-β, targeting MDSCs as a communication hub of immunosuppression may be an efficient method to abrogate the induction of multiple NK-suppressive cell types (127).

#### Regulatory T Cells

Tregs in the TME restrain antitumor NK cells. Regulatory T (Treg) cell and NK cell function are inversely correlated in multiple solid tumors including gastrointestinal stromal tumor (GIST), CRC, and prostate carcinoma (133). In cervical carcinoma, tumors were enriched for TGF-β expressing Tregs compared to peripheral blood, and Tregs were able to suppress NK cell function *ex vivo (* 134). In patients with HCC, decreased CD56^dim^CD16^+^ NK cells were found in tumor regions associated with increased CD4^+^CD25^+^ Treg infiltration (135). Tregs can inhibit NK cell function *via* multiple mechanisms. In mice, diphtheria toxin-mediated ablation of Tregs resulted in increased NK cell cytotoxicity against missing-self targets *via* increased availability of IL-2 (136). Adoptively transferred Tregs actively suppressed the ability of NK cells to control metastasis *via* TGF-β in T cell-deficient *Rag1^-/-^* mice, while Treg depletion combined with IL-12 therapy similarly boosted NK cell control of primary and metastatic mammary carcinoma (137). Multiple approaches to Treg depletion *via* low-dose cyclophosphamide, depleting antibodies, ICB, and small molecules have been trialed in preclinical and clinical studies, with varied results (138). Further progress in Treg depleting strategies may depend on improved specificity, while combination therapy with other immune-activating treatment modalities will warrant caution due to the potential for exacerbated immune adverse events.

#### B Cells

B cells are potent activators of the immune system but the role of tumor-associated B cells is poorly understood. Combined antigen receptor and scRNA-seq analysis of B cells isolated from triple-negative breast cancer compared to peripheral blood revealed that intra-tumoral B cells exhibit greater clonal diversity and more differentiated memory and plasma B cells with higher rates of somatic hypermutation (SHM) (139). The presence of germinal center, marginal zone, and class-switched memory B cells suggested the presence of active tertiary lymphoid structures (TLSs) within tumors. Additionally, higher transcript levels of CD20 and naïve and memory B cell signatures from tumor samples correlated with both improved overall survival and disease-free survival. Other studies support the role of B cells in maintaining TLSs *via* cytokine and chemokine production in inflammatory settings such as chronic obstructive pulmonary disease or in gut lymphoid structures (140–142). NK cells and B cells may engage in functionally relevant cross-talk. NK cells can enhance production of IgG and IgM by preactivated B cells and initiate early steps of B cell class switching *via* IFN-γ secretion and direct CD48-CD2 interactions (143). While NK-B cell interactions within tumors have not been well-characterized, a recent study showed that r PD-L1 and CTLA-4 expressing regulatory B cells can regulate NK cell activity (140). Further study may reveal novel pathways of NK cell reactivation, particularly in the context of TLSs.

### NK Heterogeneity and Developmental Arrest in the TME

#### Normal NK cell development

The process of NK cell development in both mice and humans is well defined (112, 144). In brief, common lymphoid progenitors (CLPs) in both species originate from hematopoietic stem cells in the bone marrow. CLPs destined to become NK cells then undergo a commitment and maturation process defined by the gain and loss of species-specific cell surface markers over time. In mice, CD122^+^ precursors gain NKG2D expression, followed by NKG2A, DNAM-1, NK1.1, NKp36/NCR1, L-selectin, and leukosialin to become immature NK cells (144). Mature NK cells express CD51, CD49b, and KLRG1, while terminal mouse NK cells go on to acquire CD43, CD11b, and expression of Ly49 subsets such as Ly49H. Mouse NK cell maturation is commonly assessed by expression of markers CD27 and CD11b, where maturing cells progress through double negative CD27^-^CD11b^-^, CD27^+^CD11b^-^, double positive CD27^+^CD11b^+^, and finally “mature” CD27^-^CD11b^+^ stages. Similarly, human NK cells undergo a maturation pathway in which CD122 expression denotes CLP commitment to the NK lineage (144). Maturing human NK cells express CD56 and gradually gain expression of NKG2D, NKp36/NCR1, NKp30/NCR3, and finally NKP80/KLRF. CD56^bright^NKp80^-^ cells express maximal levels of NKG2D, NKp36, NKp30, NKG2A, and CD161, after which they mature into a CD56^bright^NKp80^+^ inflammatory cytokine-producing state. The final stage of human NK cell maturation is traditionally thought to be the more cytolytic, less inflammatory CD56^dim^CD16^+^ state, though there is controversy regarding whether the progression from CD56^bright^ to CD56^dim^ represents a linear progression of maturation, two differing terminal stages, or plastic interchangeable NK cell states (145).

### NK Cell Development in Cancer

Multiple studies have observed NK cells in an immature and less activated state in tumors (1, 20, 21). Tumor presence has been shown to alter peripheral NK cell maturation in primary and secondary lymphoid organs (SLOs) (21). In mice, the presence of thymoma, breast, colon, and melanoma cell line-derived subcutaneous tumors led to inhibited NK cell development in the bone marrow, as evidenced by reduced mature NK1.1^+^CD11b^+^ NK cell fractions in tumor bearing mice compared to controls (21). Spleens from tumor-bearing mice also showed a decreased fraction of mature NK cells, though to a lesser degree than in the bone marrow. This peripheral maturation arrest was independent of T cell presence or bone marrow metastasis. Functionally, tumor-induced immature NK cells had decreased ability to control tumor growth *in vivo* and exhibited decreased IFN-γ production in response to *in vitro* stimulation with IL-15. Within the tumor itself, studies using PyMT mice showed that breast tumor-infiltrating NK cells in late-stage tumors exhibited an increased proportion of immature double negative CD27^-^CD11b^-^ cells compared to the spleen, suggesting that factors in the TME may inhibit NK cell maturation or cause de-maturation of mature NK cells in the local environment as well (1). These data raise the question of how spatial and temporal factors regulate tumor-induced NK cell maturation dysfunction, whether through de-maturation of mature NK cells in the TME or preferential attraction of immature NKs. Further studies will be important to understand where and when immature NK cells are generated during tumorigenesis and what combination of mechanisms is responsible, providing insight toward developing strategies of restoring intra-tumoral NK cell maturation.

In human disease, examination of metastases and lymph nodes from patients with advanced melanoma revealed that infiltrating NK cells were predominantly immature and less cytotoxic CD56^bright^CD16^-^ subsets, while mature cytolytic CD56^dim^CD16^+^ tumor-infiltrating NK cells that were present expressed lower levels of granzyme B and perforin compared to NK cells in peripheral blood (131). This suggests that the TME can affect both maturation and effector function, though the discussion surrounding the origin of CD56^bright^ versus CD56^dim^ NK cells raises the question of whether these cells are developmentally arrested or represent differential immunosuppression of two distinct terminal NK cell types. Other studies have found enrichment of CD56^bright^Perforin^low^ NK cells in breast and lung tumors, while colon cancer tissues contained an increased proportion of CD56^dim^CD16^+^ tumor-infiltrating NKs similar to melanoma (146). NK cells isolated from NSCLC tissues exhibited an inhibited phenotype, with decreased activating receptors NKp30, NKp80, CD16, NKG2D, ILT2, and DNAM-1 and increased NKp44, NKG2A, CD69; these cells were also functionally impaired compared to circulating peripheral blood NK cells based on decreased tumor killing and degranulation in tumor cell co-culture (147). Likewise, intra-tumoral NK cells from invasive breast cancer expressed less NKp30, NKG2D, DNAM-1, CD16, CD25, CD57, perforin, granzyme, and tumor necrosis factor-related apoptosis-inducing ligand (TRAIL), as well as higher NKG2A and NKp44 compared to both normal breast tissue and carcinoma in situ (20). Together, these data suggest that NK cells in a wide range of human tumor types are locally rendered immature and dysfunctional, even compared to peripheral blood NK cells from the same patient. Interestingly, increased NKp44 observed on NK cells in NSCLC and breast cancer may alternatively suggest that these cells have been activated by the TME, attempted to respond to tumor, and have now entered a state resembling immune exhaustion. Indeed, NK cells from mice exposed to continuous IL-15 stimulation for 15 days expressed decreased NKG2D, DNAM-1, TRAIL, and FasL alongside increased NKG2A, similar to the phenotype seen in patient tumors (148). However, It may also be possible that these cells represent activated immature NK cells in the TME, or even an activated phenotype of other ILC lineages. Clinically, accumulation of immature NK cells was associated with worse outcomes in HCC, suggesting that NK cell maturation status is a clinically relevant feature of cancer that merits further study (149). While the precise mechanisms that influence the observed dysfunctional cell states of intratumoral NK cells are not well understood, understanding the origins of these cells may reveal new targets for NK-specific ICB, thus prolonging the NK cell antitumor response with the potential to simultaneously enhance activation of adaptive antitumor immunity.

#### ILC Heterogeneity in the TME

NK cells represent only one type of group 1 ILC, a heterogeneous group of cell types that still remain controversial (50). Group 1 ILCs have been broadly defined by their ability to produce Th1 cytokines such as IFN-γ and encompass NK cells, tissue-specific trNKs, and non-NK ILC1s. In mice, NK cells and trNKs are defined by their expression and dependence on the transcription factor Eomes for development, while ILC1s can be broadly identified as Eomes^-^CD200R^+^. Tissue resident cells have previously been identified by markers CD69 and CD49a, though these markers can be differentially expressed during development, infection, and cancer (2). Additionally, mouse studies have shown that tissue-specific ILC1 populations may require unique developmental programs, such as Hobit-dependent liver ILC1s, further complicating study of specific ILC1 subtypes in mice (52, 150). In humans, precise identification of specific ILC subsets and their developmental origins has proven even more elusive due to limitations of studying human samples *ex vivo* and the inability to generate *in vivo* developmental knockout models (151, 152). Human group 1 ILCs have been thought to include NK cells as well as CD127(IL-7Rα)^hi^ and CD127^lo^ ILC1s. CD127^hi^ ILC1s largely inhabit the lamina propria, lack expression of EOMES or human NK cell markers, and predominantly produce cytokines, while CD127^lo^ ILC1s resemble intraepithelial CD8^+^ T cells and exhibit cytotoxic function as well as retained expression of EOMES, which could represent a subset of trNK cells (152). Similarly, unique populations of human trNKs have been identified in the liver, lung, thymus, and uterus, with some overlap with mouse ILC1 markers (47).

Recent studies utilizing high-dimensional cytometry and unbiased single-cell genomics have been invaluable in parsing out the complex diversity of tissue- and activation- specific ILCs (153). For example, an early effort to identify tissue-specific human ILC subtypes at a higher resolution using mass cytometry suggested that CD127^hi^ ILC1s could not be identified in any of the tissues examined (154). In contrast, scRNA-seq analysis of human ILCs in blood, tonsil, lung, and colon indicated the presence of *EOMES* ^-^ and *EOMES* ^+^ ILC1s in blood, tonsil, and lung. However, this data set was limited by a relatively low number of cells analyzed, and the identified ILC1 populations lacked *TBX21* expression (155). Subsequently, scRNA-seq analysis of lean and obese human adipose tissue at a higher cell number was able to distinguish ILC1s, immature, mature, and tissue-resident NK cells, with markers verified by flow cytometry (156). In this study, CD200R1 was identified as a reliable marker distinguishing ILCs from mNK in the adipose tissue. Within CD200R1^-^ NK cells, mNK, iNK, and trNK could be identified by gating on EOMES versus PERFORIN, revealing EOMES^int^PERFORIN^hi^ mNK, EOMES^lo^PERFORIN^int^ iNK, and EOMES^hi^PERFORIN^int^ trNK. Among CD200R1^+^ ILCs, ILC1s in the adipose were distinguished by expression of *ZNF683*, *TBX21*, and *CXCR6*, as well as TBET expression by flow cytometry. RNA velocity analysis revealed putative ILC developmental pathways within the adipose, identifying a unique shared ILC3 and ILC1 progenitor population. Applying unbiased genomic approaches to studying ILCs within the TME may reveal similar stage- and tissue-specific alterations to ILC development and function as well as new markers to better identify specific subpopulations and/or lineages.

Because ILC subtypes are not well characterized, studies of ILCs in the TME have been confounded by difficulties identifying true distinct cell types rather than immature or activated NK cells. One study previously suggested that the TME may directly convert conventional NK cells into a functionally impaired double-positive CD49a^+^CD49b^+^ “intermediate ILC1” phenotype (157). However, unbiased characterization of ILC development in mouse models of solid tumors using scRNA-seq revealed that these double-positive NK cells were likely not bona fide tissue-resident CD49a^+^ cells but rather TGF-β-suppressed circulating NK cells expressing CD49a (153). Another recent study using scRNA-seq was able to identify a unique Hobit-dependent developmental trajectory of ILC1s in the mouse liver from an immature stage to a cytotoxic effector stage, demonstrating that these unbiased methods may allow us to accurately distinguish between developmental stages versus distinct group 1 ILC cell lineages in the TME (51). Similar studies such as these may allow us to more accurately identify the relative contributions of different ILC subtypes to tumor control both in mouse models and human disease.

## Discussion

NK cells entering the TME encounter a rich and complex community of immunosuppressive factors. As we begin to understand the distinct mechanisms by which NK cells are regulated by their neighbors and their environment, it will be possible to identify pathways that can be directly modulated by therapeutic strategies to improve clinical outcomes. Already, multiple approaches are being tested to relieve NK cell immunosuppression in the TME. As previously mentioned, ICB blocking the PD-1/PD-L1 or CTLA-4 axis is beginning to be understood as not only acting on T cells but also antitumor NK cells. Administration of immune agonists such as CD40 ligand presents another route of promoting continued NK cell function in the TME and may synergize with other anticancer therapies (35, 158, 159). Similar to the “armored CAR” approach being applied to CAR-T cells, CAR-NK cells engineered to produce IL-15 as they persist within the TME have been tested in preclinical and clinical scenarios with some promise (160–164). Another CAR-based approach to bypassing immunosuppression utilized a chimeric extracellular TGF-β receptor domain with the intracellular activating domain of NKG2D, thereby converting a suppressive signal to an activating signal (165). Clustered regularly interspaced short palindromic repeat (CRISPR)/Cas9 editing of either autologous cord-blood NK cells or the readily-available NK-92 human NK cell line may be an alternate method of producing NK cell therapy that is intrinsically resistant to evolving immunosuppressive mechanisms over time, though this will depend on identification of key NK cell repressor molecules to be edited.

While NK cells are a comparatively rare cell type in the TME compared to other immune populations, emerging methods will enable higher resolution analysis of NK cell dynamics and ILC heterogeneity within the tumor. scRNA-seq atlases of healthy and tumor tissue have provided the resolution to identify rare and new cell subtypes (166) while advances in single cell ATAC-seq have provided insight into unique epigenetic ILC states. Evidence in T cells suggests that differential chromatin landscapes may uniquely identify immune subtypes compared to transcriptional signatures, while distinct chromatin accessibility in NK cells can distinguish between differentiated and “memory” subsets (108, 167). Combining scRNA-seq and scATAC-seq will be a powerful tool to fully survey changes in the spectrum of intra-tumoral NK cell and other ILC subset roles based on combined transcriptional and epigenetic profiles in an unbiased manner. Analysis of these data can also reveal putative cellular interactions within the TME based on predicted ligand-receptor interactions (168, 169). Once identified, combining these computationally-identified signaling partners with high-dimensional spatial validation will offer deeper insight into novel NK cell-TME interactions and the relevant signaling pathways that dictate NK development and function within tumors. Multiple groups have developed multiplex IHC and IF approaches to surveying tumor tissue samples, revealing striking clinical correlates (60). Multiplex imaging using upwards of 10 colors can be used to identify the spatial colocalization of heterogeneous cell types in the tumor and provide confirmation of cell-cell interactions computationally inferred from scRNA-seq. In addition, spatial sequencing techniques combining spatial cellular localization data with transcriptional data have the potential to inform where in the TME transcriptionally-identified cell types reside and who they interact with. While commercially-available approaches such as 10x Visium or Nanostring GeoMx do not yet provide spatial resolution down to a single cell level, recent single-cell spatial sequencing methods will prove to be a powerful tool to assess the TME once further refined (170, 171).

**Figure 3:**
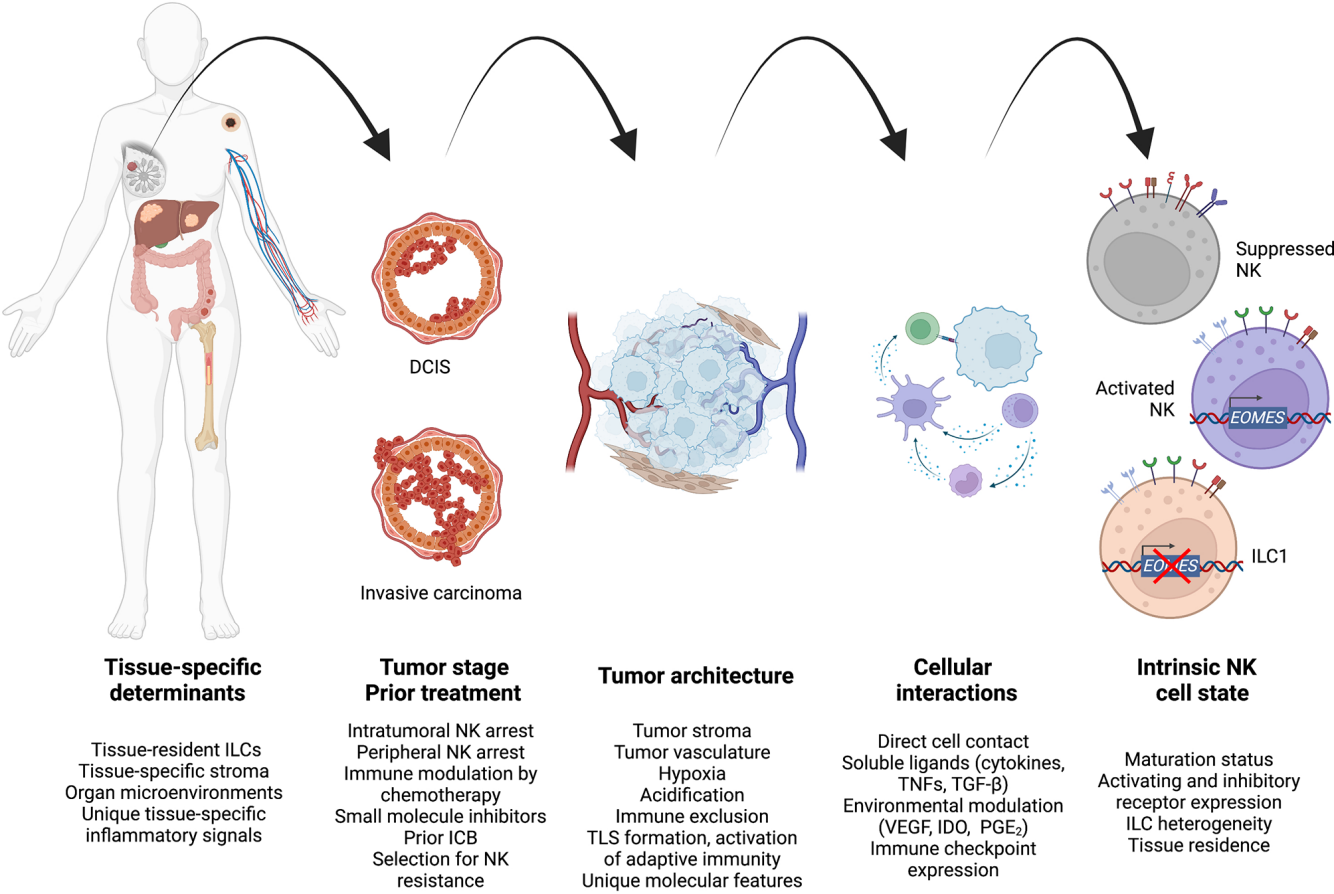
A unified model to integrate high-level spatiotemporal and intracellular factors governing antitumor NK cell regulation during cancer progression. Created using Biorender.com.

NK cell dysfunction within the TME is a progressive process that occurs heterogeneously across tissues and cancer stages (**Figure 3**). Applying new understandings of the interplay between innate and adaptive immunity may reveal novel applications of cell therapy, for example sequential NK and CAR-T cell therapy to maximize T cell activation and neoantigen presentation to the endogenous pool of antitumor T cells. At the same time, further study may allow us to identify the ideal treatment methodology to harness specific NK cell functions such as metastatic control, though long-term suppression of disease spread may require genetic modification to enhance NK cell persistence. As therapeutics specifically targeting NK cells undergo further preclinical and clinical study, studies defining how immunosuppression evolves will inform rationally optimized NK cell treatment design and treatment combinations for cancer therapy.

## Author Contributions

Both JL and TO’S planned, wrote, and revised the manuscript. All authors contributed to the article and approved the submitted version.

## Funding

This work was supported by the National Institutes of Health (P30 DK063491 and AI145997 to TO’S**)**. JL was supported by the UCLA Medical Scientist Training Program (NIH NIGMS T32GM008042).

## Conflict of Interest

The authors declare that the research was conducted in the absence of any commercial or financial relationships that could be construed as a potential conflict of interest.

## Publisher’s Note

All claims expressed in this article are solely those of the authors and do not necessarily represent those of their affiliated organizations, or those of the publisher, the editors and the reviewers. Any product that may be evaluated in this article, or claim that may be made by its manufacturer, is not guaranteed or endorsed by the publisher.
